# A survey of non-communicable diseases and their risk factors among university employees: a single institutional study

**DOI:** 10.5830/CVJA-2017-021

**Published:** 2017

**Authors:** I Agaba Emmanuel, O Akanbi Maxwell, N Okeke Edith, A Agaba Patricia, N Ocheke Amaka, M Gimba Zumnan, Daniyam Steve

**Affiliations:** Department of Medicine, University of Jos, Nigeria; Department of Medicine, University of Jos, Nigeria; Department of Medicine, University of Jos, Nigeria; Department of Family Medicine, University of Jos, Nigeria; Department of Obstetrics and Gynaecology, University of Jos, Nigeria; Department of Medicine, Jos University Teaching Hospital, Nigeria; University Health Centre, University of Jos, Nigeria

**Keywords:** diabetes mellitus, hypertension, non-communicable disease, obesity, physical inactivity

## Abstract

**Background:**

The incidence of non-communicable diseases(NCDs) is rising globally, with its attendant morbidity andmortality, especially in developing countries. This study evaluatedthe prevalence of NCDs and their risk factors amongmembers of a university community.

**Methods:**

All employees of the university were invited to the University health clinic for screening, using the World Health Organisation’s STEPwise approach to NCDs.

**Results:**

A total of 883 (521; 59.0% males) employees with a mean age of 44 ± 10 years were studied. The median (IQR) number of NCD risk factors was three (two to three) per participant. The most common NCD risk factors were inadequate intake of fruit and vegetables (94.6%; 95% CI: 92.8–95.9), physical inactivity (77.8%; 95% CI: 74.9–80.5%) and dyslipidaemia (51.8%; 95% CI: 48.4–51.6%). Others included obesity (26.7%; 95% CI: 23.9–29.8%), alcohol use (24.0%; 95% CI: 21.3–27.0%) and cigarette smoking (2.9%; 95% CI: 2.0–4.3). Hypertension was the most common NCD (48.5%; 95% CI: 45.1–51.8%), followed by chronic kidney disease (13.6%; 95% CI: 11.4–16.1) and diabetes mellitus (8.0%; 95% CI: 6.4–10.1). There was no gender-specific difference in the prevalence of NCDs.

**Conclusion:**

This study identified that NCDs and their modifiable risk factors are highly prevalent in this community. Workplace policy to support the adoption of healthy living is needed.

## Background

The incidence of non-communicable diseases (NCDs) is rising globally, with its attendant morbidity and mortality. NCDs (particularly cardiovascular disease, diabetes and cancers) were responsible for 38 million (68%) of the world’s 56 million deaths in 2012.^1^ Studies have shown that early detection and timely intervention can prevent further morbidity and ultimately prolong life. Additionally, some risk factors for these diseases, when identified, can be modified, thus preventing their onset and progression. Developing countries are currently witnessing an epidemic transition from communicable diseases to non-communicable diseases.^1^ Many individuals in these countries are caught in this ‘epidemic transition of illnesses’ as a result of lifestyle changes.

In Nigeria, the common NCDs include cardiovascular disease, hypertension, diabetes and cancers.^2^ Many studies have documented the rising prevalence of NCDs among the general population in Nigeria. Hypertension is said to affect 25 to 48% of the adult population, while nearly 10% are diabetic,^1^,^6^ and the incidence of cancer is on the increase.^3^,^4^

Recently, attention has focused on special populations, such as healthcare providers, civil servants and bankers, as they are thought to be among the relatively affluent in the community.^5^-^7^ University employees over time have also become affluent (personal communication) and therefore are also likely to be at risk of NCDs due to changes in lifestyle and increasing urbanisation. However, very few studies have addressed NCDs among university employees in Nigeria.^8^,^9^ The magnitude of NCDs and their risk factors in this subset of the population therefore largely remains unknown.

We embarked on this cross-sectional study to describe the prevalence of selected NCDs and their risk factors among the staff members of a university in north-central Nigeria. We also used this project to sensitise the participants on NCDs, as workplace interventions have been found to lead to health promotion.^10^

## Methods

A cross-sectional study of adults, aged 18 years and over employed in the University of Jos, was conducted over a fourmonth period (February to June 2014). The study was resident at the university health centre.

At the end of July 2010, the University workforce comprised a total of 2 603 people (1 793 senior and 810 junior staff). The minimum sample size (380) was calculated from the Kish formula,11 using the prevalence of hypertension (as this is the NCD with the highest prevalence) and a precision of 5%.

Sensitisation of the university staff members was carried out using invitation letters through the various directorate heads, announcements on the university FM radio station, and banners placed at strategic places such as the entrances and exits of the university and the health clinic two months prior to and during the study period.

All employees of the university who subsequently presented to the university health clinic during the study period were recruited into the study. Pregnant and menstruating women were excluded from the study as anthropometric measurements and urine testing for abnormalities would not be useable.

The Human Research and Ethics Committee of the Jos University Teaching Hospital approved the study. All participants gave written informed consent before participation. All participants had the opportunity to be counselled on healthy lifestyles, and participants found to have NCDs were referred for appropriate care.

All the participants were evaluated using a modified version of the World Health Organisation (WHO) STEPwise approach to non-communicable disease.^12^ STEP 1 entailed history taking, looking particularly for risk factors for NCDs and the lifestyle of the subjects.

STEP 2 involved a physical examination in which the height and weight were measured using an electronic weighing scale, stadiometer and non-stretch tape measure, respectively. The body mass index (BMI) was calculated from the Quetelet index.^13^ Blood pressure was measured using the OMRON digital sphygmomanometer.

STEP 3 involved obtaining blood samples for casual plasma glucose, serum creatinine, total cholesterol and high-density lipoprotein cholesterol levels, and urine testing for proteinuria and haematuria. Casual plasma glucose (CPG) level was estimated using the glucose oxidase method. Serum creatinine was assayed using the kinetic enzymatic method, and estimated glomerular filtration rate (eGFR) from the measured serum creatinine level using the CKD-EPI calculator.^14^ The laboratory analyses of the tests were carried out at the commercial laboratory of APIN, Jos University Teaching Hospital, Jos.

Generalised obesity, hypertension, diabetes mellitus and dyslipidaemia were defined according to internationally accepted guidelines.^13^,^15^-^17^ Chronic kidney disease (CKD) was regarded as the presence of proteinuria using urine dipsticks and/or eGFR < 60 ml/min/1.73 m2.^18^

## Statistical analysis

Data obtained were analysed using the Epi Info 7 statistical software (CDC, Atlanta, GA). Means ± SD were used to describe normally distributed continuous variables, and proportions for categorical variables. Median with range was used to describe non-normally distributed continuous variables. The Student’s t-test was used to compare group means and the chi-squared test to compare proportions. The Fisher exact test was used when cells contained less than five observations. The non-parametric Mann–Whitney U-test was used to compare non-normally distributed continuous variables. A p-value < 0.05 was considered significant.

## Results

A total of 883 (521; 59.0% males) employees with a slight predominance of junior-cadre workers participated in the study (Table 1). The majority were between 31 and 60 years old with a mean age of 44 ± 10 years. Women were older than the men and half had completed tertiary level education. The majority (80.5%) were married, with a median monthly household income of US$400 equivalent (US$1:00 exchanged for N150:00 as at the time of the study).

**Table 1:** Characteristics of 883 staff members of the University of Jos evaluated for select non-communicable diseases between February and June 2014

*Variable*	*Total (n = 883)*	*Males (n = 521)*	*Females (n = 362)*	*p-value*
Mean age, years	44 ± 10	43 ± 10	45 ± 9	0.002
Age group, years, n (%)^*^
< 20	3 (0.3)	2 (0.4)	1 (0.3)	< 0.0001
21–30	83 (9.4)	61 (11.7)	22 (6.1)
31–40	257 (29.1)	166 (31.9)	91 (25.1)
41–50	294 (33.3)	155 (29.8)	139 (38.4)
51–60	215 (24.3)	115 (22.1)	100 (27.6)	
> 60	31 (3.5)	22 (4.2)	9 (2.5)
Married (n = 878); n (%)	707 (80.5)	437 (84.2)	270 (75.2)	< 0.0001
Tertiary education completed (n = 876); n (%)	440 (50.2)	243 (46.9)	197 (55.0)	0.02
Junior staff (n = 843); n (%)	466 (55.3)	319 (63.0)	147 (43.6)	< 0.0001
Monthly income, USD, median	400	333.33	466.66	< 0.0001
BMI (kg/m2)	27.2 ± 5.1	25.1 ± 3.5	30.2 ± 5.7	< 0.0001
SBP (mmHg)	129 ± 19	130 ± 19	127 ± 20	0.06
DBP (mmHg)	79 ± 12	79 ± 12	80 ± 11	0.4
CPG, median (mg/dl)	85.0	85.0	86.0	0.10
[mmol/l]	[4.72]	[4.72]	[4.77]	
Proteinuria (n = 883) (%)	116 (13.2)	72 (13.8)	44 (12.2)	1.15
Serum creatinine (mmol/l)	74.5 ± 19.3	81.8 ± 19.7	64.0 ± 13.1	< 0.0001
eGFR (ml/min/1.73m2)	114.2 ± 20.5	115.1 ± 20.7	113.1 ± 20.2	0.15
Reduced eGFR	4 (0.4)	2 (0.4)	2 (0.5)	0.69
TC (mg/dl)	193.4 ± 43.9	201.4 ± 46.2	187.9 ± 41.4	< 0.0001
[mmol/l]	[5.01 ± 1.14]	[5.22 ± 1.20]	[4.87 ± 1.07]
HDL-C (mg/dl)	56.6 ± 16.4	60.7 ± 16.5	53.8 ± 15.7	<0.0001
[mmol/l]	[1.47 ± 0.42]	[1.57 ± 0.43]	[1.39 ± 0.41]

^*^Fisher exact test; USD: United States Dollars; BMI: body mass index; SBP: systolic blood pressure; DBP: diastolic blood pressure; CPG: casual plasma glucose; eGFR: estimated glomerular filtration rate; TC: total cholesterol; HDL-C: high-density lipoprotein cholesterol.

The median (IQR) number of NCD risk factors was three (two to three) per participant. The most common NCD risk factors were inadequate intake of fruit and vegetables (94.6%; 95% CI: 92.8–95.9), physical inactivity (77.8%; 95% CI: 74.9– 80.5%) and dyslipidaemia (51.8%; 95% CI: 48.4–51.6%). Details of NCD risk factors by sociodemographic variables are shown in Table 2.

**Table 2:** Sociodemographic characteristics and distribution of non-communicable diseases and their risk factors among 883 staff members of the University of Jos, Nigeria

*Variable*	*Tobacco % (95% CI)*	*Alcohol % (95% CI)*	*Diet % (95% CI)*	*Physical inactivity % (95% CI)*	*Obesity % (95% CI)*	*Dyslipidaemia % (95% CI)*	*HPTN % (95% CI)*	*DM % (95% CI)*	*CKD % (95% CI)*
All	2.9 (2.0–4.3)	24.0 (21.3–27.0)	94.6 (92.8–95.9)	77.8 (74.9–80.5)	26.7 (23.9–29.8)	51.8 (48.4–51.6)	48.5 (45.1–51.8)	8.0 (6.4–10.1)	13.6 (11.4–16.1)
Gender
Male	4.8 (3.2–7.1)	33.8 (29.8–38.0)	94.6 (92.2–96.3)	72.9 (68.9–76.7)	10.6 (8.1–13.6)	48.8 (44.4–53.1)	43.0 (38.7–47.4)	6.9 (5.0–9.5)	14.2 (11.4–17.6)
Female	0.3 (0.0–1.8)	9.9 (7.2–13.6)	94.5 (91.5–96.5)	84.8 (80.7–88.3)	50.0 (44.7–55.3)	55.0 (39.8–50.3)	56.4 (51.1–61.5)	9.7 (6.9–13.3)	12.7 (9.5–16.7)
Marital status
Married	3.0 (1.9-4.6)	24.3 (21.2-27.7)	94.6 (92.6-96.1)	79.5 (76.3-82.4)	27.0 (23.8-30.5)	53.2 (49.4-56.9)	49.4 (45.6-53.1)	8.8 (6.8-11.2)	13.0 (10.7-15.8)
Unmarried	2.8 (0.9–6.5)	22.7 (16.8–29.6)	94.3 (89.8–97.2)	71.0 (63.7–77.6)	25.6 (19.3–32.7)	46.0 (38.5–53.7)	44.9 (37.4–52.6)	5.1 (2.4–9.5)	15.9 (10.8–22.2)
Age group, years
< 20	0.0 (0.0–70.8)	0.0 (0.0–70.8)	100.0 (0.0–29.2)	100.0 (0.0–29.2)	0.0 (0.0–70.8)	33.3 (0.8–90.6)	33.3 (0.8–90.6)	0.0 (0.0–70.8)	0.0 (0.0–70.8)
21–30	7.2 (2.7–15.1)	7.2 (2.7–15.1)	88.0 (79.0–94.1)	73.5 (62.7–82.6)	7.2 (2.7–15.1)	36.1 (25.9–47.7)	14.5 (7.7–23.9)	2.4 (0.3–8.4)	7.2 (2.7–15.1)
31–40	3.1 (1.4–6.0)	22.2 (17.3–27.8)	94.9 (91.5–97.3)	69.3 (63.2–74.8)	22.2 (17.3–27.8)	42.4 (36.3–48.7)	36.2 (30.3–42.4)	3.9 (1.9–7.0)	15.6 (11.4–20.6)
41–50	2.0 (0.8–4.4)	29.9 (24.8–35.3)	96.9 (93.8–98.4)	80.6 (75.6–85.0)	29.9 (24.8–35.5)	53.4 (40.8–52.5)	49.0 (43.1–54.8)	5.8 (3.4–9.1)	12.6 (9.0–16.9)
51–60	2.3 (0.8–5.3)	34.9 (28.5–41.7)	93.5 (89.3–96.4)	85.6 (80.2–90.0)	34.9 (28.9–41.7)	65.1 (58.3–71.5)	71.2 (64.6–77.1)	16.7 (12.0–22.4)	14.4 (10.0–19.8)
61–70	3.2 (0.1–16.7)	32.3 (16.7–51.4)	96.8 (83.3–99.9)	77.4 (58.9–90.4)	32.3 (16.7–51.4)	64.5 (45.4–80.8)	80.6 (62.5–92.5)	19.4 (7.5–37.5)	19.4 (7.5–37.5)
Education, years
None	0.0 (0.0–60.2)	0.0 (0.0–60.2)	0.0 (0.0–60.2)	0.0 (0.0–60.2)	0.0 (0.0–60.2)	25.0 (0.6–80.6)	75.0 (19.4–99.4)	0.0 (0.0–60.2)	0.0 (0.0–60.2)
< 7 years	6.3 (2.1–14.0)	31.3 (21.3–42.6)	97.5 (91.3–99.7)	85.0 (75.3–92.0)	32.5 (22.4–43.9)	52.5 (41.0–63.8)	63.8 (52.2–74.2)	17.5 (9.9–27.6)	13.8 (7.1–23.3)
8–11 years	3.3 (0.7–9.2)	29.3 (20.3–39.8)	95.7 (89.2–98.8)	84.8 (75.8–91.4)	23.9 (15.6–33.9)	46.7 (36.3–57.4)	48.9 (38.3–59.6)	7.6 (3.1–15.1)	8.7 (3.8–16.4)
> 12 years	2.7 (1.6–4.3)	22.0 (18.9–25.5)	94.0 (91.8–95.7)	76.3 (72.7–79.5)	26.4 (23.1–30.1)	52.7 (48.7–56.6)	45.8 (41.8–49.7)	6.6 (4.9–8.9)	14.3 (11.7–17.3)
Staff cadre
Junior	4.4 (2.9–6.8)	25.9 (22.1–30.0)	94.5 (92.1–96.3)	77.2 (73.2–80.7)	20.8 (17.4–24.7)	57.7 (52.4–62.4)	43.2 (38.8–47.7)	5.7 (3.9–8.2)	12.9 (10.2–16.3)
Senior	1.0 (0.3–2.8)	21.6 (17.7–26.2)	94.6 (91.7–96.5)	78.6 (74.2–82.6)	34.3 (29.6–39.3)	47.3 (42.8–51.8)	55.2 (50.1–60.2)	11.1 (8.2–14.7)	14.4 (11.2–18.4)
Income quintile
Lowest	3.4 (1.1-7.9)	28.4 (21.2-36.5)	93.0 (87.6-96.6)	75.0 (67.1-81.8)	20.1 (13.9-27.6)	43.1 (34.8-51.6)	38.1 (30.2-46.6)	4.9 (2.0-9.8)	10.4 (5.9-16.6)
Second	6.1 (2.9-10.9)	28.6 (21.8-36.2)	93.2 (88.3-96.6)	81.7 (74.9-87.3)	20.1 (14.2-27.0)	48.8 (40.9-56.7)	40.2 (32.6-48.1)	4.9 (2.1-9.4)	15.8 (10.6-22.3)
Third	0.0 (0.0-100.0)	22.3 (15.8-30.1)	94.4 (89.2-97.5)	81.1 (73.7-87.1)	33.5 (25.8-41.9)	53.8 (45.3-62.2)	52.4 (43.9-60.8)	9.1 (4.9-15.0)	13.9 (8.7-20.7)
Fourth	3.0 (1.0-6.9)	18.2 (12.7-25.0)	95.7 (91.4-33.0)	82.9 (76.2-88.3)	25.6 (19.1-33.0)	57.3 (49.4-65.0)	56.1 (48.1-63.8)	11.6 (7.1-17.5)	11.5 (7.1-17.5)
Fifth	0.6 (0.02-3.5)	22.7 (16.3-30.1)	95.4 (90.8-98.1)	76.6 (90.8-98.1)	35.0 (27.5-43.1)	59.7 (51.5-67.6)	55.1 (46.9-63.2)	11.0 (6.6-17.1)	13.6 (8.6-20.0)

HPTN = hypertension; DM = diabetes mellitus; CKD = chronic kidney disease.

**Fig. 1:**
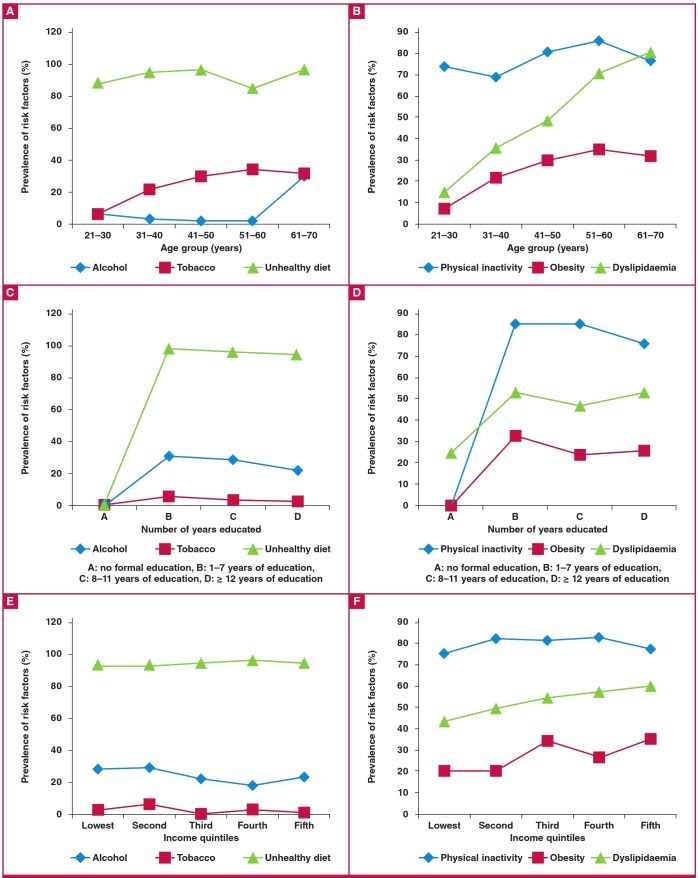
Prevalence of non-communicable disease risk factors in relation to some sociodemographic characteristics in 883 staff members of the University of Jos.

No participant admitted to passive (second-hand) smoking at home or in the work environment and none used smokeless tobacco. As shown in Fig. 1, tobacco use (Fig. 1A), obesity and dyslipidaemia (Fig. 1B) increased with age.

A low intake of fruit and vegetables was common in participants with a formal education (Fig. 1C), as were physical inactivity, obesity and dyslipidaemia (Fig. 1D), compared to those without formal education. Fig. 1F shows that physical inactivity and dyslipidaemia increased with increasing household income.

**Fig. 2:**
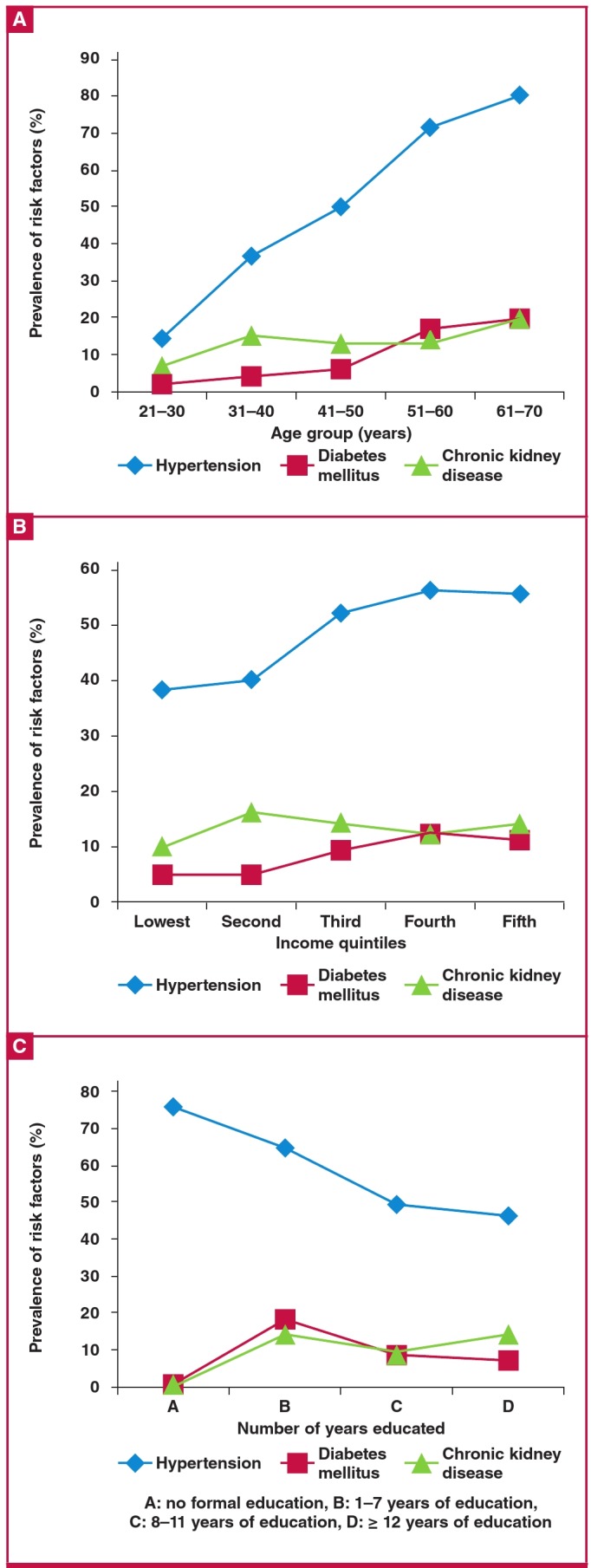
Prevalence of non-communicable diseases in relation to some sociodemographic characteristics among 883 staff members of the University of Jos.

Hypertension was the most common NCD, being present in nearly half the participants (48.5%; 95% CI: 45.1–51.8%), as indicated in Table 2. Its prevalence rose with increasing age (Fig. 2A) and household income (Fig. 2B) but decreased with increasing level of education (Fig. 2C). Similar trends were noticed for diabetes mellitus (DM) with regard to age and household income (Fig. 2A, B). CKD also increased with increasing age (Fig. 2A). The prevalence of DM and CKD by sociodemographic characteristics is shown in Table 2 .

## Discussion

The main findings of our study were that: (1) the most prevalent NCD risk factors were low intake of fruit and vegetables, physical inactivity and dyslipidaemia, with the majority of participants having multiple factors; (2) nearly half (48.5%) of the participants were hypertensive, and CKD and DM occurred in 13.6 and 8.0%, respectively; and (3) the sociodemographic characteristics of age, income and education impacted on the prevalence of the common NCDs and their risk factors.

Inadequate consumption of fruit and vegetables was the most prevalent risk factor in this study, followed by physical inactivity and obesity. This is in accord with the findings of many researchers. Sufficient consumption of fruit and vegetables was lacking in 96.6% of respondents in a Nepalese community.^19^ Zaman and co-workers^20^ reported that 92% of participants in a nationally representative sample in Bangladesh reported inadequate intake of fruit and vegetables (median serving of 1.6 portions/day).

In a study of university employees in western Nigeria, 96% of participants had inadequate consumption of fruit and vegetables.^8^ Similarly, a high prevalence (90%) has been reported from northern Nigeria.^6^ However, in a community survey in eastern Nigeria, a slightly lower proportion (70.4%) of respondents had inadequate intake of fruit and vegetables.21 Nonetheless, a high prevalence of inadequate intake of fruit and vegetables exists in the general population and that needs to be addressed.

Physical inactivity has been found to contribute significantly to NCD-related mortality.^22^ Three-quarters of the participants in our study were physically inactive. This is in accord with the findings of Oladimeji and co-workers,^6^ who reported that 91% of workers in the public sector were physically inactive. Likewise, nearly 80% of hospital workers in Nigeria have been reported to be physically inactive.^23^ However, a lower prevalence of physical inactivity has been reported in earlier studies.

Of the 2 000 persons studied in Togo, 41% were sedentary, while 35% was reported from Bangladesh.^24^,^25^ In a study among workers at a medical college in Ghana, only 25% were physically inactive.^26^ The reason for the disparity between our findings and those of prior studies reporting low prevalence of physical inactivity may be related to the highly selective nature of our study participants.

We noted the rarity of both active and passive cigarette smoking in our participants. This is in keeping with previous reports that document a paucity of smoking among Nigerians.^8^,^6^,^27^ Generally, this finding is in contrast to the findings in southern Africa, Asia^20^,^28^ and the Western world,^29^-^31^ where smoking constitutes a major public health hazard.

Clustering of risk factors was prevalent in this study, with the median number of risk factors being three (IQR 2–3) per participant. This finding corroborates the findings of previous studies. In a study of over 3 800 South African adults aged 50 years and above, Phaswana-Mafuya and associates^32^ reported a mean incidence of risk factors of three. In a recent German survey, 45.1% of participants had multiple risk factors.^33^ Similar clustering has been reported by the SAGE wave 1 study that evaluated older adults across six countries.^34^

A study among Senegalese private sector workers revealed that more than half of the participants had two or more cardiovascular risk factors.^35^ Villegas and co-workers^36^ reported that 67.6% of men and women sampled across 17 general practice settings in Ireland had more than one cardiovascular risk factor. This scenario is the typical clustering in patients and deserves attention to reverse or limit their contribution to NCD and its related mortality.

The prevalence of the selected NCDs parallels that obtained in the literature from the Western world and the African region. Hypertension was present in nearly half of the participants; CKD was present in a little over a 10th of the population, and DM in nearly a 10th. In the SAGE wave 1 study, the prevalence of hypertension ranged from as low as 17.9% in Bangladesh to as high as 78% in South Africa among older persons.34 A prevalence of 47.2% was reported among Irish hospital attendees in a study that evaluated over 1 000 patients recruited from several general practices.

Oluyombo and colleagues,37 working in south-west Nigeria, reported a prevalence of 47.2% among residents of a semi-urban community. A slightly lower prevalence of 31.4% was recently reported from south-east Nigeria.^21^ In a large community survey that evaluated 5 206 adults in Malawi, Msyamboza and associates^38^ reported a prevalence of 33% among persons aged 25 to 64 years. A recent review by Bosu^7^ demonstrated that the prevalence of hypertension among workers in the West African sub-region has steadily increased from 12.9% in the 1980s to 37.5% in 2014, while figures up to 51.6% (95% CI: 49.8–53.4) and 43% (95% CI: 42.1–43.9) have been recently reported in Nigeria among urban and rural populations, respectively.^4^

CKD, an emerging NCD, has gained attention in recent times as it is both an end-point of communicable and non-communicable diseases and a strong cardiovascular risk factor. It has become a pandemic, affecting both developed and developing countries. CKD was present in a significant proportion of the participants in our study. Similar reports exist regarding the prevalence of CKD from the Western world and Asia.^39^-^41^,

However, varying reports from the African region exist. In a recent community survey from Senegal that studied 1 037 adults, CKD was present in 4.9% of the participants.42 In a similar study from Cameroun, the prevalence of CKD ranged from 11.0 to 14.2%, depending on the prediction equation used.^43^ In a study that evaluated 402 private sector IT workers in Dakar, Senegal in late 2010, 22.4% had CKD.^35^ The prevalence of CKD in Nigeria in various subsets of the population has been reported to range from 7.8% among public sector employees,44 to 11.4% in the community45 and 43.5% among retirees,46 depending on the criteria used.

The prevalence of DM in this study parallels the estimated global prevalence of 9%, the WHO estimated prevalence of 7.9% in Nigeria in 2014,1 and the 9.7% recently reported from Senegal.^35^ It is however slightly lower than the 11% obtained among university employees in south-western Nigeria.^8^ However, our study differed from theirs as they relied on selfreported diagnosis, which is subject to recall bias. Oluyombo and associates^37^ recently reported that 6.8% of 750 respondents had DM. Our finding together with the foregoing support the assertion that the prevalence of DM is on the increase in Nigeria. However, the prevalence of DM in our study was higher than the 2.5% reported by Oladapo and co-workers^47^ in south-west Nigeria, and the 3.6% by Okpechi and colleagues^21^ in southeastern Nigeria.

That sociodemographic characteristics impact on NCDs and their risk factors was confirmed by the findings of our study. The prevalence of hypertension, CKD and DM rose with increasing age, as expected. Their prevalence also increased with increasing income, as a result of the concomitant rise in the prevalence of some of the risk factors with increasing income. It is noteworthy that hypertension decreased with increasing educational level. This confirms the results of prior studies that reported an inverse relationship between educational level and hypertension.^19^,^48^ This provides an opportunity for intervention in order to halt the rising trends in NCD.

Together with the existing literature, our study has implications for the subset of employees at this university and the general population at large, as large numbers of these individuals are at an elevated risk of NCD-related events. In a recent review of national policies addressing NCDs in low- and middle-income countries, Lachat and colleagues^22^ demonstrated the disconnect that exists between the burden of NCDs and the response of the respective governments, including Nigeria. Concerted efforts are needed to stem the high prevalence of NCDs and their risk factors in our environment, so as to achieve the 2025 voluntary global targets of the Global NCD Action Plan.^1^

## Limitations

The findings of this study must be interpreted within the limitations inherent in the study design. We studied only employees of the university hence the generalisability of the findings is limited. The purposive sampling process used may also have introduced selection bias in the study. A stratified systematic sampling would have yielded a more representative sample. However we invited all the staff members of the university to participate in the study.

We were unable to measure triglyceride levels so we used non-fasting blood samples for the determination of lipid levels. At first glance, one may assume that assessing lipid abnormalities using casual plasma samples (and not in the fasted state) as we did in this study would constitute a limitation. However, the lack of effect of fasting on levels of serum total cholesterol and reduced high-density lipoprotein cholesterol has been documented and therefore casual plasma sampling is used in field studies.^49^,^50^

We were also unable to repeat proteinuria assessments or eGFR after three months and therefore the prevalence of CKD may have been spuriously high. Finally, we could not establish causality as our study was cross-sectional in design. Despite these limitations, we have studied the largest sample of university employees in Nigeria to date. Our study therefore provides the fulcrum for further studies of this nature to elucidate the burden of NCDs in this category of workers.

## Conclusion

This study identified that the most prevalent NCD risk factors among employees of a university are behavioural and therefore modifiable. We also demonstrated that the NCDs and their risk factors are impacted upon by sociodemographic characteristics. Given the burden of NCDs and their risk factors among this subset of the general population, there is a need for workplace policies aimed at health promotion to be put in place in order to stem the rising trend of NCDs. Multicentre studies addressing the burden of NCDs among university employees are imperative.
